# “The Road Less Traveled”: Endovascular Embolization of a Type II Endoleak via Corona Mortis

**DOI:** 10.3390/diagnostics16081195

**Published:** 2026-04-16

**Authors:** Nicolò Rossini, Laura Maria Cacioppa, Alessandro Felicioli, Luca Felici, Vincenzo Vento, Marzia Rosati, Pietro Boscarato, Roberto Candelari, Chiara Floridi

**Affiliations:** 1 Division of Interventional Radiology, Department of Radiological Sciences, University Politecnica delle Marche, 60126 Ancona, Italy; nicolorossini44@gmail.com (N.R.); alessandro.felicioli@ospedaliriuniti.marche.it (A.F.); marzia.rosati@ospedaliriuniti.marche.it (M.R.); pietro.boscarato@ospedaliriuniti.marche.it (P.B.); roberto.candelari@ospedaliriuniti.marche.it (R.C.); c.floridi@staff.univpm.it (C.F.); 2 Department of Clinical, Special and Dental Sciences, University Politecnica delle Marche, 60126 Ancona, Italy; 3 Department of Cardiovascular Sciences, University Hospital “Azienda Ospedaliera Universitaria delle Marche”, 60126 Ancona, Italy; lucafelici9995@gmail.com (L.F.); vincenzo.vento@ospedaliriuniti.marche.it (V.V.); 4 Division of Radiology, Department of Radiological Sciences, University Hospital “Azienda Ospedaliero Universitaria delle Marche”, 60126 Ancona, Italy

**Keywords:** corona mortis, type 2 endoleak, common iliac artery aneurysm, ethylene vinyl alcohol copolymer, endovascular embolization, anatomical variant

## Abstract

Type 2 endoleaks (EL2s) are potentially life-threatening complications, defined as persistent arterial perfusion of the excluded aneurysmal sac after endovascular aneurysm repair (EVAR). Most EL2s are managed endovascularly, through embolization of the aneurysmal sac and its arterial feeders. During embolization, attention should be given to anatomical variants such as “corona mortis”, an arterial anastomosis connecting external iliac (via inferior epigastric) and internal iliac (via obturator) arteries. We present the case of an 88-year-old male previously treated with EVAR for a left common iliac artery aneurysm (CIAA), complicated by EL2 originating from the ipsilateral ilio-lumbar branch of the internal iliac artery. Successful embolization of the endoleak was achieved through catheterization of the inferior epigastric artery, taking advantage of the “corona mortis” variant. This route allowed access to the sac and embolization with ethylene-vinyl-alcohol-copolymer. This approach represents a safe alternative to direct sac puncture or superior gluteal artery access in patients exhibiting this anatomical variant.

**Figure 1:**
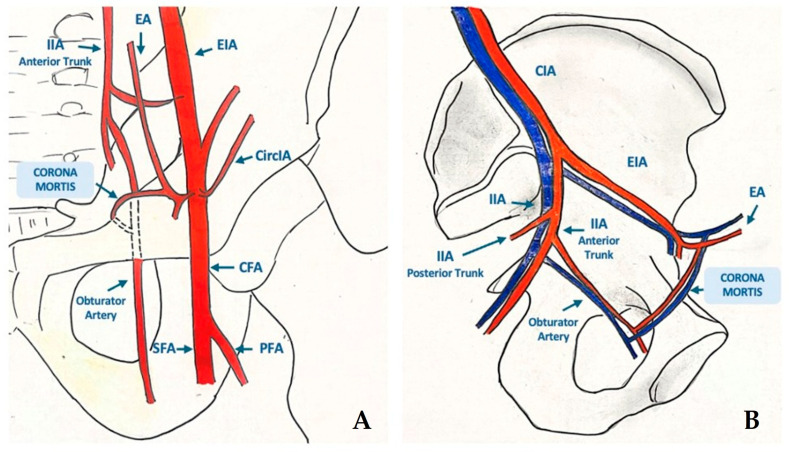
Common iliac artery aneurysms (CIAAs) often coexist with abdominal aortic aneurysms, whereas isolated cases are rare, accounting for 0.4–1.9% of intra-abdominal lesions. Although typically asymptomatic and incidentally detected, they carry a rupture risk of ~5%, and up to 18% present with symptoms. Due to their deep pelvic location, symptoms including local visceral or venous compression occur when IAAs reach considerable size. Endovascular aneurysm repair (EVAR) is currently the first-line approach for CIAAs, offering lower perioperative mortality, fewer complications, and shorter hospital stays. EVAR includes internal iliac artery (IIA) embolization and deployment of a covered stent graft from the common to external iliac artery, while branched prostheses can preserve IIA patency [1]. Type 2 endoleaks (EL2s) following EVAR of CIAAs typically occur due to reperfusion of the excluded sac via the IIA and its collateral branches [2,3,4]. In standard arterial anatomies, treatment options involve percutaneous sac puncture or endovascular access via the superior gluteal artery [5,6,7,8]. However, in the presence of anatomical variants, alternative endovascular routes may be considered to safely reach and embolize the aneurysmal sac [9,10,11]. We present a case of endovascular treatment of an EL2 via femoral access in a patient with “corona mortis”. This anatomical variant is an arterial anastomosis between the external iliac artery (via the inferior epigastric branch) and the IIA (via the obturator branch), running posterior to the superior pubic ramus, within the Retzius space (Figure 1) [12,13,14]. Until now, the “corona mortis” arterial anastomosis has been considered relevant mainly in the context of pelvic trauma [12,13]. Catheterization and navigation through this anastomotic vessel provided selective access to IIA and its iliolumbar branch, responsible for the EL2. Unlike previously reported cases, in which alternative approaches such as direct sac puncture or gluteal artery access are most commonly described, this case highlights the technical feasibility of a fully transarterial approach through the “corona mortis”, enabled by a stepwise catheterization strategy under combined fluoroscopic and cone-beam CT (CBCT) guidance, allowing superselective catheterization of the feeding iliolumbar branch in a post-EVAR setting. In addition, a successful use of ethylene-vinyl-alcohol copolymer as an embolic agent in this uncommon access route was described. “Corona mortis” anatomy on antero-posterior (**A**) and lateral (**B**) views: arterial (and possible venous) anatomical variant consisting in an anastomosis between the external iliac artery (EIA), through the inferior epigastric artery (EA) and the internal iliac artery (IIA), through the obturator artery. This anastomosis runs posterior to the superior pubic ramus, within the Retzius space (CircIA = circumflex iliac artery; CFA = common femoral artery; PFA = Profunda femoral artery; SFA = superficial femoral artery).

**Figure 2:**
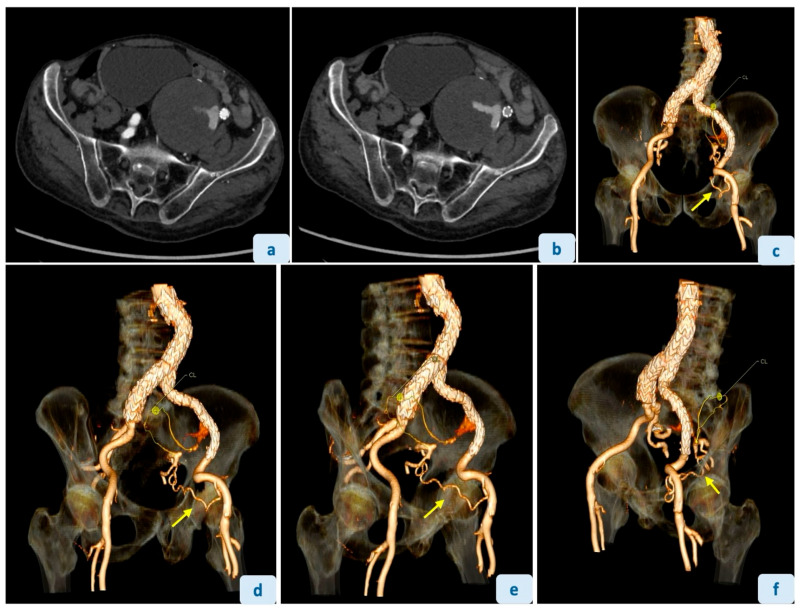
An 88-year-old male with a history of open aorto-aortic graft for ruptured abdominal aortic aneurysm (2021) and subsequent development of a left CIAA treated with EVAR (2023), presented to our Emergency department with diffuse abdominal pain and vomiting, raising suspicion of intestinal subocclusion. Computed tomography angiography (CTA) confirmed a large left-sided CIAA, increased in size compared with 2023 imaging (84 vs. 61 mm in axial diameter), without signs of rupture or fissuring, involving the entire length of the CIA up to the origin of the IIA. The increase in size was attributed to the presence of an EL2 originating from the ipsilateral iliolumbar artery (**a**,**b**) with a tortuous course (yellow thin line with “CL” mark) (**c**–**f**). A careful review of the CTA identified a “corona mortis” variant (yellow arrow), with reperfusion of the excluded IIA (**c**–**f**). The case was discussed in a multidisciplinary meeting, including vascular surgeons. Although the patient’s abdominal pain was not attributable to the CIAA, the indication for treatment was based on significant aneurysmal sac enlargement (>2 cm in maximum diameter over approximately 2 years of follow-up), according to current European Society for Vascular Surgery (ESVS) guidelines [1]. The patient was considered not eligible for surgical repair due to age and comorbidities and was referred to our Interventional Radiology department for endovascular management. Given the prior exclusion of the IIA by the endograft, the “corona mortis” anastomosis provided a direct arterial pathway to access both the IIA and the iliolumbar branch supplying the aneurysmal sac.

**Figure 3:**
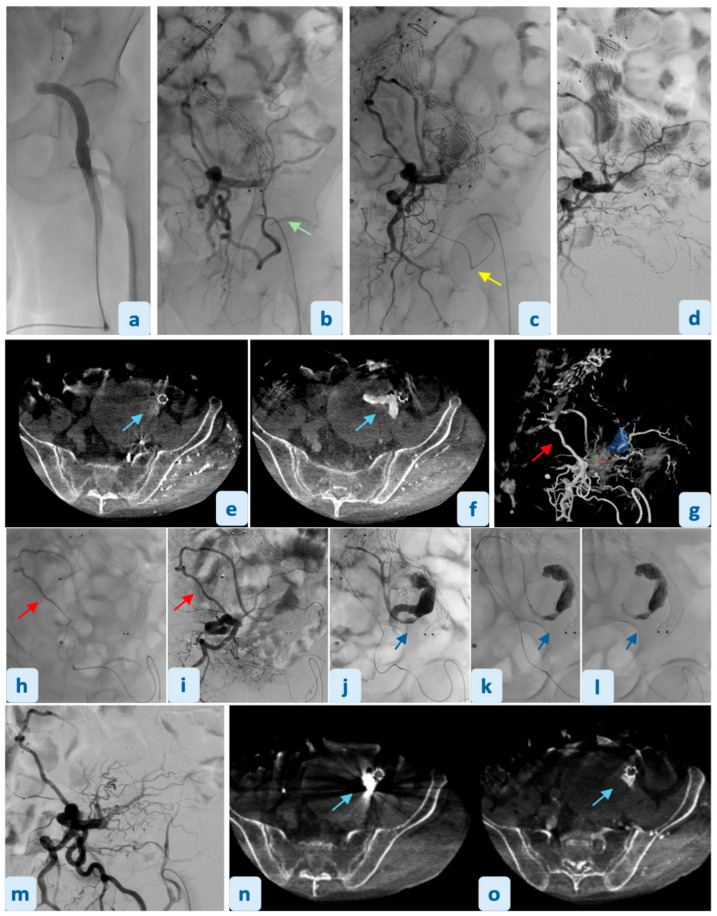
The procedure was performed in our Institutional angiosuite (Azurion Xper FD20, Philips Healthcare, Amsterdam, The Netherlands). Left retrograde low femoral arterial access was obtained and a 4 Fr introducer sheath was introduced (**a**). The left inferior epigastric artery was catheterized (green arrow) using a Berenstein 1 catheter (**b**). A 2.7-Fr microcatheter system (Progreat 150 cm, Terumo Medical, Tokyo, Japan) (yellow arrow) was advanced into the proximal tract of the anastomotic vessel (**c**). A superselective angiographic overview confirmed the communication with the left obturator and internal iliac arteries (**d**). Given the vessel tortuosity, the initial system was replaced by a Cobra C1 supporting catheter (Radifocus^®^ Glidecath^®^, Terumo, Tokyo, Japan) and a Transend^®^ Floppy 0.014 guidewire (Boston Scientific, Boston, MA, USA) to reach and navigate the IIA. Selective angiography and catheter-directed dual-phase contrast-enhanced cone-beam CT (CBCT) confirmed an EL2 (light blue arrow) supplied from the left ilio-lumbar artery (red arrow) (**e**–**g**). Successful catheterization of the markedly tortuous iliolumbar artery (red arrow) enabled microcatheter advancement into the aneurysmal sac, with subsequent sac angiography confirming EL2 perfusion (blue arrow) (**h**–**j**). Embolization was performed using a single 6 mL vial of ethylene-vinyl-alcohol-copolymer (Onyx^®^ 34L, Covidien, Galway, Ireland). The injection was performed under fluoroscopic monitoring until an occlusive cast was achieved within the sac and the distal tract of the iliolumbar artery (blue arrow) (**k**,**l**). Onyx-34 was selected due to its controlled, non-adhesive and progressively solidifying properties, allowing deep and homogeneous penetration of the aneurysmal sac and distal feeding iliolumbar branch. Onyx was preferred over coil embolization, which may be limited in small-caliber and tortuous vessels due to suboptimal packing and risk of incomplete occlusion. Final catheter-directed angiography demonstrated complete exclusion of the aneurysmal sac with no residual flow from the iliolumbar artery (**m**). Non-contrast CBCT overlaid with pre-embolization contrast-enhanced CBCT confirmed adequate distribution of the embolic agent (light blue arrow) (**n**,**o**). The procedure was performed with a total procedure time of 2 h 13 min. Total fluoroscopy time was 57 min 37 s. Radiation exposure metrics included a cumulative dose area product (DAP) of 204,529 µGy·m^2^ and a total air kerma of 1045 mGy. The patient was discharged after two days without post-procedural complications, and a 1-month CTA confirmed complete exclusion of the aneurysmal sac. Endovascular management of EL2s originating from IIAs in patients treated with EVAR represents a technical challenge for interventional radiologists due to limited transarterial access options and the need for meticulous pre-procedural planning. Direct sac puncture remains a widely used technique, offering direct access to the aneurysmal sac; however, it may be associated with risks such as bowel or graft injury and requires prone positioning, which may limit its applicability in frail patients [6,15]. The transgluteal approach via the superior gluteal artery represents another established alternative; nevertheless, it can be technically challenging due to deep pelvic location, increased procedural complexity, and potential risk of non-target embolization [5,15,16,17]. While both these techniques are established approaches, this case report describes an alternative route feasible only in the rare anatomical variant of the corona mortis. This approach offers several technical advantages, including supine patient positioning, avoidance of anatomical risks associated with direct sac puncture, and easier access and hemostasis compared with superior gluteal artery puncture [15,16,17]. However, this treatment strategy may be limited by the tortuosity and unfavorable angulation of the corona mortis, the difficulty of identifying this anatomical variant pre-procedurally, the small vessel caliber, and the risk of unintentional injury of adjacent branches [18,19,20]. Potential failure scenarios include inability to identify or catheterize the anastomosis, excessive vessel tortuosity preventing distal navigation, or inadequate delivery of embolic material to the target branch. Therefore, this approach should be considered highly operator-dependent and currently applicable only in selected cases with favorable anatomical conditions and advanced expertise. In conclusion, the “corona mortis” may represent a feasible, safe and effective transarterial pathway for the treatment of EL2 in selected patients with this anatomical variant. Although technically demanding due to vessel tortuosity and small caliber, this approach may broaden the spectrum of the available endovascular options when conventional routes such as direct sac puncture or gluteal artery access are challenging or not feasible. Careful pre-procedural imaging evaluation and thorough knowledge of materials remain essential to ensure a tailored, efficient, and safe intervention.

## Data Availability

The original contributions presented in this study are included in the article. Further inquiries can be directed to the corresponding author.
